# Nutritional Value of Silkworm Pupae (*Bombyx mori*) with Emphases on Fatty Acids Profile and Their Potential Applications for Humans and Animals

**DOI:** 10.3390/insects14030254

**Published:** 2023-03-03

**Authors:** Mihaela Hăbeanu, Anca Gheorghe, Teodor Mihalcea

**Affiliations:** Research Station for Sericulture Baneasa, 013685 Bucharest, Romania

**Keywords:** *B*. *mori*, silkworm pupae, fatty acids, nutritive value

## Abstract

**Simple Summary:**

The nutritional composition of silkworms will likely have multiple implications for humans, animals, and the environment. The silkworm pupae attracted interest due to lipids and protein profiles. Furthermore, the valuable level of the essential fatty acids (alpha-linolenic and linoleic from the n-3 and n-6 family) results in significant physiological functions in the human body that support good health. Using silkworm pupae to feed humans and animals reduces the waste that must be discarded; however, this is only one way of lowering pollution emissions. On the other hand, as a unique silkworm feed, mulberry leaves have the capacity to sequester carbon by absorbing a significant amount of harmful pollutants.

**Abstract:**

*Bombyx mori* is an ideal lepidopteran species representative of many scientific studies, a model of studies for medicine and a significant insect from an ecological standpoint. This review was performed to summarize the fatty acids (FA) composition of silkworm pupae (SP) that are associated with other important compounds that could add value to SP, diversifying the ways of valorization. The proposal to complete plant-based feeds with insect-based feeds represents a viable option to beneficially impact human and animal health and the environment. The quality and quantity of fats consumed significantly impact the aetiology of certain diseases. The key compounds of fat named essential FA (EFA) substantially influence the prevention and treatment of several diseases through their nutraceutical functions. Due to its excellent profile in nutrients such as protein and fat, amino acids and fatty acids composition, SP has become an important alternative feed ingredient and source of EFA. SP is a by-product that was discarded in large quantities. Following the need to act to improve human health and reduce climate change impact, many researchers focused on studying SP applications in the medical and agricultural industries. Several authors noticed an improvement in the health markers by using SP. The feed cost for the animal was reduced with economic implications. Minimization of environmental impact was recorded. Few precautions were recommended regarding SP use, although they should not be ignored. The composition of SP and its potential for use in various industries provides us with persuasive arguments for continuing to develop the sericulture industry.

## 1. Introduction

The quality and quantity of fats consumed significantly impacts the aetiology of certain diseases. Numerous specialists have focused on recommended guidelines for the quantity and type of fat to consume. The primary constituent of lipid structure is straight-chain aliphatic carboxylic acids, known as fatty acids (FA). The most natural FA range from C4 to C22 [1]. Medical and scientific studies demonstrated the effectiveness of polyunsaturated FA (PUFA) in preventing and treating several diseases, including improved insulin sensitivity, reduced blood pressure, decreased thrombotic tendency, anti-inflammatory and antiarrhythmic effects, improved vascular endothelial function, and increased plaque stability [2,3,4]. PUFA act through receptors that regulate various cellular and metabolic processes, including adipogenesis, inflammation, oxidative stress, and the metabolism of lipids and glucose connected to energy homeostasis [5]. 

PUFA are mainly composed of n-6 (linoleic acid, C18:2n-6) and n-3 (alpha-linolenic acid, C18:3n-3), from which derive other long-chain PUFAs [6]. Our body lacks the necessary enzymes to synthesize PUFAs with important implications for life and thus they must be received from the diet. Essential fatty acids (EFAs), respectively alpha-linolenic and linoleic FA, have been regarded as nutraceuticals and functional foods [6]. On the other hand, n-3 FA has gained attention as a possible preventive and therapeutic agent to lower the risk of certain diseases due to its influence on the pathways involved in atherosclerosis and myocardial infarction.

Plant-based edible oils are becoming increasingly well-liked today due to their usefulness and health-enhancing qualities [4,7]. Vegetable oils, which include all saturated FA (SFA), monounsaturated FA (MUFA), and PUFA families, are sources of essential FA (EFA). However, the source plant or the technological procedure used to extract oil determines the content of their FA. Most of the investigated vegetable oils have higher PUFA content, notably higher n-6 PUFA concentration; however, some have a higher composition in beneficial n-3 PUFA, such as linseed, hempseed, camelina, pumpkin and sesame oil etc. [6,8,9,10,11,12]. The primary issues are related to the low worldwide production and ineffective technologies for processing edible oil from plants [13]. 

A sustainable alternative source of n-3-rich PUFA, which is recommended to be considered, is silkworm pupae (SP). Besides the excellent protein content of SP (about 55.6% as dry matter basis, DM), the fat concentration (about 32% as DM basis) can have higher importance. SP is considered a safe and valuable source of beneficial n-3 PUFA. As a result, SP oil is viewed as a reliable source with a variety of applications, such as in the food and pharmaceutical sectors, having anti-inflammatory action or stimulant effect on lymphatic circulation, and can be used in medicine as a treatment of sinusitis, otitis, bronchitis, asthma, tuberculosis, urinary infections, and post-surgery, and can also have possible cosmetics applications [14]. Both n-3 and n-6 FA, present in SP oils as primary components, are crucial for treating diabetes and cardiovascular disorders. 

**Figure 1:**
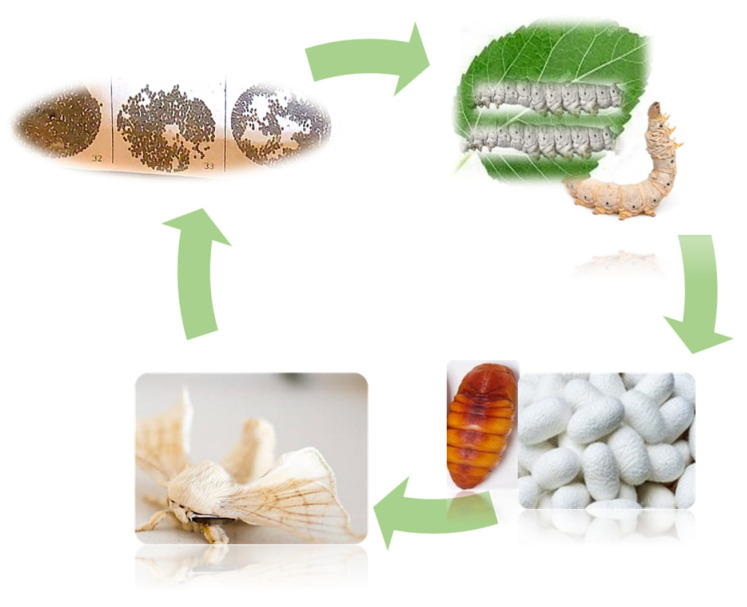
The life cycle of silkworm *B*. *mori*.

*Bombyx mori* (*B. mori*) is an excellent lepidopteran species representative for numerous scientific investigations, one of the most important insects from an economic point of view, but also as a model organism for medicine that is utilized in many developing nations. More than 3000 strains have been created and kept up over the lengthy period of domestication. According to Mokaya et al. [15] *B. mori* produce about 90% of the world’s silk. The silkworm is characterized by a short and rapid life cycle with four stages (egg, larvae, cocoon/pupa, and adult moth), as seen in Figure 1.

In Romania and other European countries, the most frequently used and raised mulberry silkworm is also the subject of extensive research. However, *Antheraea pernyi*, currently used primarily as a source of insect food and for cosmetic purposes, and *Philosamia ricini* (*P. ricini*)*,* recognized for its white or brick-red eri silk, which is widely used in Brazil, China, and India, are important as well. In light of these considerations, this review focused on *B. mori*, aiming to summarize the FA composition of SP and their importance that associated with other bioactive compounds could influence SP value and diversify the ways of valorization and impact the environment.

## 2. Data Sources

This paper reviewed publications from online scientific articles available on PubMed, Web of Science, MDPI, Elsevier, Springer, Wiley Online Library, Research Gate, and Google Scholar databases to provide FA composition of SP and opportunities to valorize the higher content in EFA in varied fields. When browsing, we used as keywords “fatty acids,” “silkworm pupae oil”, “*B. mori*”, “plant edible oil”, “pupae nutritive value”, and “composition and applications”. A shortlist of 82 special interest articles was selected, analyzed, and presented. The publications were screened for nutritional composition, FA profile and their beneficial role in the body, and applications for humans and animals, with few exceptions, considering a time window of publications between 2010 and 2023. Another criterion of selection was the language of the papers. We took into consideration only papers in the English language. The articles were split into two groups: one with reported nutritional value, especially FA composition and beneficial effect, and another focused on potential applications. 

The software IBM SPSS version 20.0 (SPSS Inc., Chicago, IL, USA) ANOVA test was used to analyze and describe statistically the potential influence of time and sex on SP FA concentration.

## 3. Nutritional Value of Silkworm Pupae 

For both people and animals, edible insects are an excellent source of nutrients, such as FAs, as the principal source of energy, proteins containing almost all amino acids, lipids, as the most important nutrient after protein and their FAs composition, minerals, and vitamins [16]. Chitosan and chitin (not cytotoxic compounds) are the two main categories of sugars found in SP that possess specific biofunctional properties and are the basis for the pharmacological actions along with other separated, refined polysaccharides that are all biologically active [17].

Furthermore, due to their sustainability, environmental friendliness, and nutritional value, insects are becoming increasingly popular as a source of protein [18]. A high dietary, therapeutic, and commercial value is associated with silkworms and their metabolites [19]. The pupae are a highly nutrient-dense waste recommended as an edible by-product from the silkworm, which is left after reeling silk [20,21]. 

The nutrients of SP are received and converted from mulberry leaves. According to all authors studied [19,21,22,23], the most predominant nutrient in DM of SP is protein, considered a complete nutrient due to the profile of essential amino acids [19]. It is known that proteins are considered a functional source of nutrients in the food sector. In addition, a total lipid content of 25–32.2% in DM was reported by numerous authors [19,23,24,25]. The calorigenic components such as protein, fat, and carbs contained by silkworms provide up to 230 kcal per 100 g. Furthermore, silkworms provide 43.6 calories with 35 g of protein. The SP contains about 55 g of protein, while the requirement for males is about 53 g/day and for females 45 g/day. According to Roychoudhury and Mishra [26], 100 g of dried eri silkworms supply 100% of the daily requirements for many vitamins (pyridoxine, riboflavin, thiamine, ascorbic, and folic acids), minerals (calcium, iron, and phosphorus), and 75% of the average individuals’ daily protein needs. 

**Figure 2:**
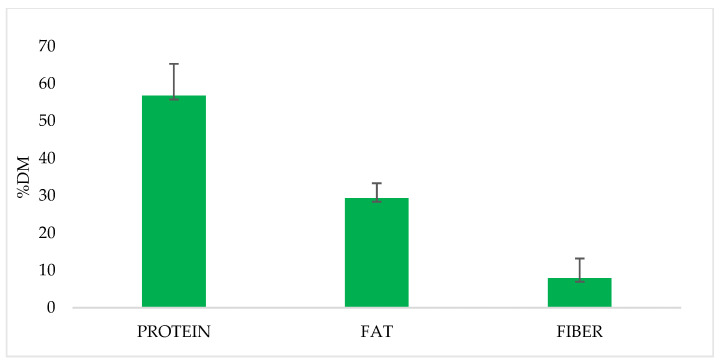
Mean and standard deviation (SD) values of main nutrients content of SP (%DM).

As indicated by several researchers, the nutritional value of SP is shown in Table 1, and average values and standard deviations are shown in Figure 2. According to Meyer-Rochow [27], there is a large variation in the nutrient content of different species of insects that can be explained by the geographic and climatic conditions as well as the feeding regimen. As seen in Table 1, for *B. mori* there were variations as well. Thus, protein contents ranging from 48 to 94.98% DM (SD 8.52), lipid values are between 12.3 and 35.7% DM (SD 3.95), and fibre content variation is between 3.5 and 14. (SD 5.21). The possible explanations of these variations could be related to the mulberry leaves’ composition, feed intake, digestibility coefficients, the bioavailability of nutrients, methods of analyses, sampling, etc. 

Several authors highlighted the quality of SP protein and the excellent composition of amino acids [19,24,35,36]. Insect proteins are typically highly digestible, estimated between 46 to 96% [16,19]. In the gut of SP are functional peptides that simulate the enzymatic digestions. These peptides may have antibacterial, anti-inflammatory, antioxidant, and immune system-modulating properties [37]. 

Zhou and Han [38] mentioned that SP contains 18 amino acids, mainly methionine, known as limitative amino acids for poultry and pigs. SP have an amino acid score of 100 and a protein digestibility-corrected amino acid score of 86, respectively [14]. 

According to Jeyaprakashsabari and Aanand [36], the amino acids composition of SP (expressed as g/16 g N) consists of alanine 5.6–5.8, arginine 5.8–5.6, aspartic acid 10.4, cystine 1.0, methionine 3.5, lysine 7.0, isoleucine 5.1, leucine 7.5, phenylalanine 5.1–5.2, threonine 5.2–5.1, tryptophan 0.9, glutamic acid 13.9, histidine 2.6, proline 5.2, serine 5.0, glycine 4.8, tyrosine 5.9, and valine 5.5.

The amino acids composition expressed as g% g protein, described by Kumar et al. [23], Zhou and Han [38] are methionine 4.6, lysine 7.5, aspartic acid 10.9, threonine 5.4, serine 4.7, glutamic acid 14.9, proline 4.0, glycine 4.6, alanine 5.5, cystine 1.4, valine 5.6, isoleucine 5.7, leucine 8.3, tyrosine 5.4, phenylalanine 5.1, histidine 2.5, and arginine 6.8.

Up to 25 different types of minerals can be found in silkworm pupae, and some of these minerals may have physiological effects on the organism. According to Zhou et al. [17], depending on the type of pupa and the location in which silkworm larvae are grown, the type and concentration of minerals in pupae can differ. SP is an excellent calcium, potassium, iron, and zinc source, positively affecting human health. Phosphorus (474 mg/100 g DM), magnesium (207 mg/100 g DM), calcium (158 mg/100 g DM), iron (26 mg/100 g DM), zinc (23 mg/100 g DM), chromium (1.69/100 g DM), manganese (0.71 mg/100 g DM), copper (0.15 mg/100 g DM), minerals were also determined in *B. mori* [17]. It is important to note that the natrium: potassium ratio in SP is very low. Heavy metals are bellow the maximum recommendations level. Consuming SP may reduce the risk of certain diseases, such as stroke, hypertension, cardiovascular disease etc. Selenium can be elevated in certain pupae. Selenium-rich SP is crucial in the fight against oxidative stress and cancer prevention [17].

Certain vitamins, such as Vitamin A (273.99 µg), Vitamin E (51.45 IU/kg), and Vitamin C, were also determined in SP by Kumar et al. [23]. Wu et al. [19] reported a content of 0.07 mg/100 g SP Vitamin B1, 2.23 mg/100 g Vitamin B2, 2.2 mg/100 g Vitamin B3 and 9.89 Vitamin E. No Vitamin A was determined. Vitamin B2 can be essential to avoid the effects caused by a vitamin B2 deficiency. Five tocopherols (α-Tocopherol, β-tocopherol, γ-tocopherol, γ-tocotrienol, and σ-tocopherol) are also present in SP.

## 4. Silkworm Pupae Fatty Acids Composition 

Oil extracted from SP represents an important by-product with an excellent FA profile. From 100 g dried SP, approximately 25–30 g oil can be extracted, depending on extraction processes [22]. According to Wu et al. [19], the lipid concentration of SP is about 13% (on a fresh basis). Kouřimská and Adámková [25] found 8.5–15 g of fat in 100 g insects. It was reported by Kotake-Nara et al. [39] that the lipid content of SP differed by sex, respectively 4.8% for males and 9.0% for females (on a wet basis).

Several literature data referred to Eri (*P. ricini*) SP because the oil content of this species is higher than in *B. mori* [14,40,41]. According to Tomotake et al. [24] and Mahesh et al. [42], SP is a good source of alpha-linolenic FA, known as valuable and functional FA. 

As shown in Table 2, the following FAs were mentioned in the literature for SP lipid content: from the SFA class were noticed the C16:0 (palmitic) and C18:0 (stearic) FAs; from MUFA class, the C16:1n-7 (palmitoleic) and C18:1n-9 (oleic) FA were identified, while in PUFA family the C18:2n-6 (linoleic) and C18:3n-3 (alpha-linolenic) were found to be more predominant. 

More than 60–70% of the oil extracted from SP constitutes unsaturated FA [22,24,44]. Among these, 43.6% are PUFA, with alpha-linolenic acid accounting for a considerable part of this (29–40.7% of total FA), while MUFA represent about 27.7%, with oleic acid accounting for the greater part (26% of total FA). Palmitic and oleic FAs have values close to alpha-linolenic. Some traces of C14:0 (myristic) and C20:3n-3 (eicosatrienoic) were also determined. Additionally, Yu et al. [45] highlighted that when silkworms were fed with fish oil as a supplement, which contained C20 and C22 PUFAs, larvae accumulated these PUFAs in proportion to the amount of fish oil supplied to the diet. However, for synthesizing these FAs, the presence of genes encoding Δ5 and Δ6 desaturases is necessary, which means a transfer of these kinds of enzymes can occur by supplementing the silkworm diet with a source rich in C20 and C22 PUFA. Thus, according to Yu et al. [45], the dietary addition of fish oil to silkworm, the eicosapentaenoic (EPA) and docosahexaenoic (DHA) were found.

**Figure 3:**
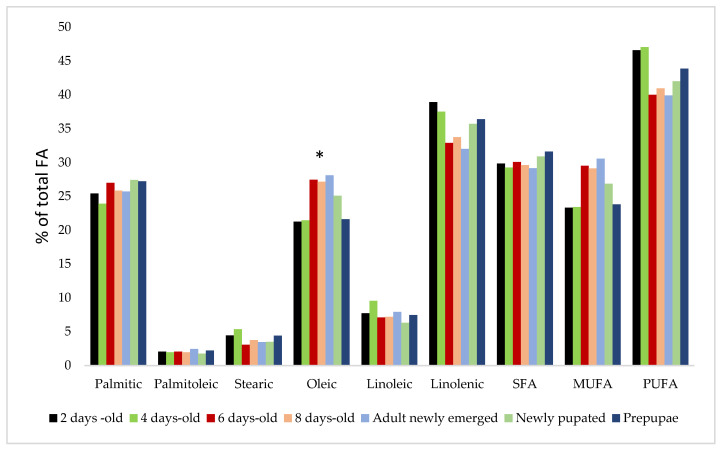
Average FA concentrations in SP depend on the stage of development, nonsignificant effect except for oleic FA, * *p* < 0.05 significant difference between means for oleic FA. The other FAs identified did not differ significantly when the stage of development was considered.

On the other hand, Nakasone and Ito [43] showed that the FA composition changed significantly during the SP development cycle only for oleic FA (Figure 3). The higher concentration of oleic FA was noticed for male adults newly emerged (29%), and the lower was seen in females two days old. If we refer to the entire SP cycle of development, the PUFA (alpha-linoleic and linolenic FA) recorded the higher value (42.91%), followed by SFA (palmitic and stearic FA, 30.06%) and MUFA (palmitoleic and oleic, 26.64%). 

**Figure 4:**
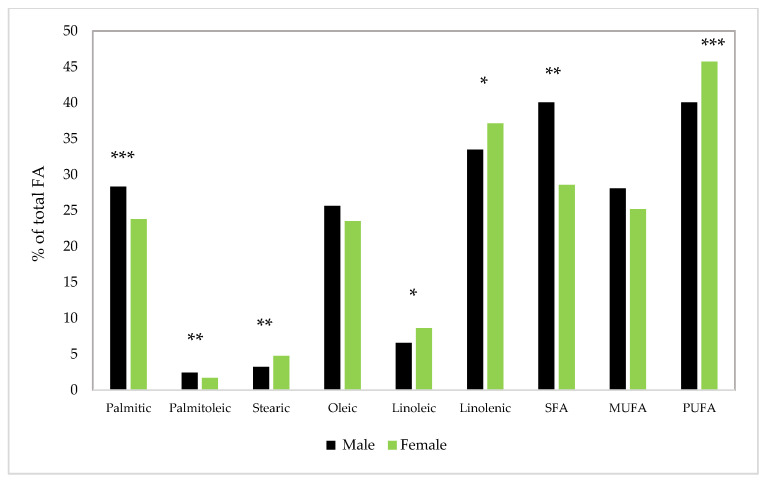
Average FA concentrations in SP throughout the entire developmental cycle, depending on sex; nonsignificant effect for oleic and MUFA FA; * *p* < 0.05 significant difference between means; ** *p* < 0.01 and *** *p* < 0.001 highly significant difference between means.

On the contrary, except for oleic and ΣMUFA FAs, sex influence the FAs concentration (Figure 4). Hence, sex has a significantly higher effect on palmitic, palmitoleic, stearic, SFA, and PUFA FAs (*p* <0.001), as well as a significant impact on linoleic and linolenic FAs (*p* < 0.05).

Whilst during the entire cycle of development, PUFA concentration was higher in females vs. males (>12.45%), SFA and MUFA observed a higher value in males (>9% and 10%, respectively). Irrespective of the sex, the most predominant was alpha-linolenic FA, which is extremely important from a human health viewpoint. The females recorded a higher concentration of alpha-linolenic FA vs. males (>9.8%). 

## 5. Role of Fatty Acids

FAs are essential molecules that control cellular communication and intracellular signaling and contribute to the fluidity of plasma membranes. Since their discovery, FAs have gained recognition for their critical functions as adjuvants in the delivery of drugs and as treatments for various ailments. From a dietary standpoint, FAs are mostly ingested as lipids, which are esterified forms of various FAs and organic alcohols such as cholesterol, sphingosine, or glycerol. 

Fats, including EFAs, play a crucial role in energy metabolism and other body processes. EFAs are the most important fats since living organisms are not able to synthesize them. EFAs are required for biological activity but can only be found in plant-based foods. Thus, alpha-linolenic and total n-3 FAs, linoleic and total n-6 FAs are indispensable and must be given through feed [46]. The enzymes required to convert omega-6 FA into omega-3 FA or vice versa are not identified within the body. Therefore, diets must contain the proper ratio of these essential fats for growth and development, as well as for good health and appropriate physiological functions; the n-6:n-3 ratio indicated by Park et al. [47] to be close to 1–2:1. The tissues in mammals are unable to insert double bonds at the n-6 and n-3 positions. However, the EFA can be converted into other longer-chained FAs and serve as potential mediators to regulate physiological processes. The EPA is produced via the conversion of alpha-linolenic acid. Once more, EPA can be transformed into DHA, albeit the rate of alpha-linolenic acid conversion into EPA is only 5–10% efficient and is blocked by linoleic acid. However, since they can be synthesized from alpha-linolenic, EPA and DHA are not included in EFA. 

Maintaining a suitable EFA level is essential for human and animal health. EFA meet a variety of functions in living organisms, including synthesizing prostaglandins, leukotrienes, cellular membranes, phospholipids, retinal photoreceptors, cerebral grey matter (brain tissue), and sperm [48]. 

The desilked SP (*B. mori*) oils were shown to be a good source of alpha-linolenic FA, which has received a lot of interest for medical and nutritional uses [49]. 

EFAs act in the body via multiple mechanisms. To induce their physiological implications on cell and tissue activity, EFAs may affect hormone and/or metabolite concentrations, which may then affect cell and tissue behavior. Another way consists of the influence of certain variables, such as LDL oxidation and oxidative stress, direct effects on cell behavior via fatty acid “receptors” or “sensors” on the cell surface or inside the cell, and effects on cell behavior mediated by changes in the make-up of cell membrane phospholipids [2]. 

## 6. Potential Applications of Silkworm Pupae 

Sericulture explores possibilities for the manufacturing of important biomaterials as well as utilization in regenerative medicines, tissue engineering, medical materials, drug delivery systems, cosmeceuticals, and food additives. Large amounts of waste are produced from the primary and secondary activities in the agro-industrial supply chain. The principal waste from the sericulture industry is SP, obtained after reeling the silkworm cocoons. These by-products can be discarded by the textile and feed, and food industries. Large-scale pupae disposal can have detrimental environmental effects in regions where silk is produced. 

SP, the most valuable by-product, gave rise to the possibility of applications in medicine, nutrition, cosmetics, animal feed, fertilizer, etc. 

Ordoñez-Araque et al. [16] proposed a change in mentality by adopting insect-based feeds instead of plant-based feeds as a first step to minimizing the environmental impact that traditional animal-based diets indirectly produce. This led as well to a considerable decrease in feed costs.

### 6.1. Application for Humans

Since the importance of insects as the final untapped biological resource began to be reconsidered in recent years, the insect industry’s expansion has been accelerating. SP, due to its high nutritional value, have the potential to positively affect health by fortifying human and animal diets with valuable bioactive compounds. 

Although the protein has a higher concentration in DM, the oil structure also recommends this by-product for use in the medical and food industries. Numerous metabolic-related diseases have been connected to increased cellular oxidation, blood glucose, and blood pressure. The intracellular reactive oxygen species (ROS) regulate several signaling pathways against inflammation to activate an immune response. An imbalance of ROS can damage cells, leading to cardiovascular disease. The production of ROS is positively correlated with PUFA levels [50]. FAs regulate many different physiological processes [51]. The vital role of PUFA in treating a wide range of illnesses and disorders, such as diseases of the central nervous system, hypertension, inflammatory and immune disorders, depression and neurological dysfunction, and visual function, has been extensively discussed in the literature [17,51,52,53,54]. The EFAs content of SP is bioactive compounds associated with polyphenols, amino acids, and other nutritional compounds that have effects of antioxidant, anti-apoptotic, anti-genotoxic, anticancer, cardiovascular protective, and hepato-protective properties [17]. The pupae have gradually undergone additional processing to extract minerals and active substances, and they are now utilized in food modification and pharmaceutical investigations [55]. 

The pharmacological functions of SP oil were well summarized by Zhou et al. [17], who evaluated several research studies. Numerous biological actions of SP oil have been linked to improved blood circulation. By enriching SP oil with sodium salt solution through an esterification and saponification process, Kim et al. [56] demonstrated a beneficial effect as a meal and medication against vascular problems. Two different insects, *Hermetia illucens* (*H. illucens*, black soldier fly)*,* fed with leftover fruits and vegetables and *B. mori*, fed mulberry leaves, were studied by Saviane et al. [57] for their oil antibacterial characteristics. They found an antibacterial effect with the same effectiveness, probably due to UFAs, typical of the SP. Related to what Saviane et al. [57] mentioned, the FAs have been found to impact Gram-positive bacteria, whilst very few species of Gram-negative bacteria are sensitive to FAs. This supports the theory that FAs are likely involved in the action mechanism of the oils. Long-chain UFAs, such as oleic, linoleic, and linolenic acids, have more antibacterial activity than SFA, such as palmitic and stearic acid, which are less effective. The antibacterial properties involve UFA in the defense system protecting against numerous pathogens to treat bacterial infections, with a positive impact on health.

Long-chain UFAs are prevalent in *B. mori* oil, mostly taken from the vegetal source used as a substrate for the larvae’s rearing. These FAs are also in higher amounts in *H. illucens* oil, particularly oleic acid. Furthermore, UFA are believed to have the ability to limit fat storage by speeding lipid metabolism. Its growing impact on fat metabolism-related proteins suggests that it can be used as a nutritional supplement.

The resulting increased lipid metabolism might also be suggested as a dietary supplement to prevent metabolic disorders [58]. Supplementing the human diet with SP oil may be an effective way to treat acute liver injury induced by an overdose of acetaminophen [59]. By adding SP oil to the diet, the area of gastric ulcer and secretion decreases; however, it raises gastric pH [60]. The oil content of SP ameliorates oxidative damage and reduces inflammations. It seems possible that eating SP could prevent Alzheimer’s disease due to the action of substances with antioxidant characteristics that decrease malondialdehyde concentration in the hippocampus. Inhibition of acetylcholinesterase can occur as well [61]. Certain peptides or amino acids play antioxidant functions. SP may have neuroprotective benefits by activating antioxidant enzymes and the cholinergic system. Therefore, SP may have therapeutic and preventative functions for neurodegenerative disorders [62]. It has been demonstrated in vitro the tyrosinase inhibitory, and free radical scavenging activities of oils and sericin of SP extracted from native silkworms (*B. mori*) [63]. 

Nutritionists claim that because SP are rich in calcium and phosphorus, two nutrients crucial for children’s growth, this food will be able to shield them from rickets and malnutrition.

Chitin present in SP skin (approximately 4% of DM) can be transformed into several beneficial substances, including chitosan, chitin sulfate, chitin nitrate, chitin xanthate, and sodium carboxymethyl chitin [32]. Chitin and chitosan derivatives are used in wound dressing, controlled release of drugs, and contact lenses. After low-temperature crystallization, chrysalis oil from SP is similar to linseed oil [32,64].

Protein hydrolysates from SP decreased nitric oxide production. A few proteins in SP hemocytes and hemolymph were found to have anti-inflammatory effects by lowering nitric oxide production [18].

An antigenotoxic activity of SP was mentioned by Deori et al. [65]. The presence of polyphenolic groups and FA-like linoleic acid may explain this.

Excreta have been used therapeutically in traditional Asian medicine to cure infectious disorders, headaches, and abdominal pain and reduce LDL cholesterol and blood pressure [66]. Silkworm excreta could have strong antioxidant activity due to its abundance of flavonoids, chlorophyll, alkaloids, carotenoids, and lutein components.

As a precaution, it should be highlighted that the SP of *B. mori* can produce certain anti-nutrients such as phytate, phytic phosphorus, as well as tannic acid, alkaloid, flavonoids, saponin, and oxalate. These anti-nutrients are present but at low concentrations acceptable to humans. As a result, humans can consume SP [19]. With regards to allergens, the WHO and International Union of Immunological Societies [67], quoted by Wu et al. [19], has not officially confirmed or recognized any allergens of SP (www.allergen.org). However, allergic reactions when SP was included in the diets were previously mentioned [19,68,69,70].

### 6.2. Application in Animal Feeding

The first mention of insects as animal feed dates to 1919, although their widespread usage began between 1960 and 1970 [16].

The protein with a higher biological value led to the opportunity of using SP in the livestock sector. Insects can only substitute the protein and fat typically added to animals’ diets. The ability to separately manipulate insect protein, different amino acids, and fat and add them in the right amounts to the feedstock might provide advantages to the feed sector. After extraction of oil, the cake remaining can be an alternative rich-protein, less expensive feedstuff for animal diets, especially for fish, poultry, and pigs [22,71], although, as Asimi et al. [72] described, only a minor part was used (about 25–30%). 

The classical protein-rich feedstuffs for monogastric are soybean meal, characterized by a higher n-6 FA content; while SP contains a higher level of n-3 FA, close to linseed [9,10,73] and camelina [74]. Several researchers [75,76] have shown that the SP can potentially replace 50% of the classical protein source, respectively, fish meal or soybean meal (5–10% dietary inclusion). 

According to Priyadharshini et al. [77] and Miah et al. [78], the dietary addition of SP did not affect the growth parameters or carcass traits of broiler and laying hens. However, the desired ratio of n-6 to n-3 is maintained if we speak in terms of meat quality. Although a complete replacement is typically possible, broilers are more susceptible to performance issues. In the same way, SP can adversely affect the capacity of growing quails to digest nutrients, primarily because of the presence of chitin and 1-DNJ [29]. 

Hens egg quality and yolk color can also be ameliorated using SP in the diet [77]. Although the important quantity of oil in SP can restrict the use of calves, the dietary addition of SP for cattle and monogastric has also been utilized mainly in Asian countries [71]. 

Priyadharshini et al. [77] obtained a higher deposition of fat and fur growth by including SP in rabbits’ feeds. 

Additionally, fish meal was substituted entirely with SP in the fish diet by Shakoori et al. [79], with good results. The results showed that SP could stimulate the immune system in rainbow trout while inducing some anaemia-related symptoms. 

SP oil reduced inflammation and oxidative stress in mice [60].

### 6.3. Environmental Effects

As was already indicated, one way to reduce the current issues arising from climate change and the environmental impact the world faces today is to employ insects for human and animal consumption. About 36% of global emissions of greenhouse gases (GHG) come from the agricultural sector, of which 78% are generated from cattle. The main GHG generated are methane (43%), nitrous oxide (29%), and carbon dioxide (27%) [16]. Depending on the species and production method, the obtaining of animal proteins has a significant negative influence on the environment. Animals generally require enormous land areas for breeding throughout their lives, as well as food resources (which impact the environment), water, fossil fuels, storage and packaging, etc.

Compared to livestock production, growing insects use less space and water and have a smaller negative impact on the environment and the economy. Insects are suggested as an alternative to reduce resource use. The edible fraction of insects is 80% to 90%. According to Ordoñez-Araque, [16], insects use 20 m^2^ to produce 1 kg of protein, 20 L water for 1 g of protein, 1 kg feed for 1 kg live weight, and 1 g average GHG/kg mass gain.

Fresh SP are a highly degradable by-product that pollutes the environment and gives off an unpleasant odour in the nearby locations. Large-scale pupae disposal can have significant environmental consequences in places that produce silk. According to Giacomin et al. [80], the mulberry is considered the starting point of the sericulture industry because it produces many of the leaves that silkworm larvae eat and has the capacity to sequester carbon. Culturing mulberry trees helps absorb a significant amount of harmful pollutants. Chemically derived fertilizers are not frequently used; 8.4 g of silk is equivalent to one mulberry tree. Mulberry fields reduce CO_2_ equivalent at about 735 times the weight of synthetic silk fiber per cultivated area.

Based on the hypothesis that adding fat-rich ingredients to the diet can reduce the amount of CH_4_ produced, we can assume that the SP could mitigate the main GHG. However, there is no evidence in the literature that nutritional strategies are more effective in decreasing the enteric CH_4_ level [11]. On the other hand, it was specified that PUFA inhibits the population of rumen protozoa that produce H_2_. It is well known that high H_2_ concentrations, produced by fermentation processes, cause a low redox potential and enhance propionate synthesis, which increases in the oil-supplemented groups [81]. About 9–25% of the rumen methanogens are associated with protozoa. Decreasing methane emissions is influenced by rumen protozoa [82]. Eliminating protozoa from the rumen (defaunation) has been shown to reduce methane emissions by 9–37% [82].

According to Sheikh et al. [71] and Thirumalaisamy et al. [81], one of the less expensive options for methane mitigation may be SP; this non-conventional oil source is utilized for human consumption in some countries. A better strategy to avoid waste and minimize the environmental impact of the silk industry also consists of using these valuable by-products to feed animals and poultry. In their study, Thirumalaisamy et al. [81] mentioned that sheep’s daily methane emissions were reduced by 23–26% over time by supplementing SP oil (2% of DM intake) on a daily or bimonthly basis.

Another strategy for sustainable development is to redirect agricultural wastes from pollution-producing applications and instead use them to make biofuels [66].

## 7. Conclusions

There was a preponderance of datasets regarding the great relevance of PUFA, emphasizing EFAs for both human and animal health. It became clear that SP might be used in both human and animal diets because of their great EFA composition and our growing understanding of the crucial role that EFAs play in maintaining overall health. Large quantities of SP are discarded by-products but attracted attention due to their nutritional potential. The numerous and complex medical effects, including immunomodulatory, antibacterial, anticancer, antioxidant, hepatoprotective, antifatigue, and anti-apoptotic functions, deserve to be mentioned here. In addition to improving the blood vessel environment, lowering blood pressure, preventing thrombosis and arteriosclerosis, increasing cell vitality, and strengthening the body’s defenses, SP bioactive compounds also reduce plasma triglycerides and can be used to treat wounds and regulate blood sugar levels. Transferring Δ5 and Δ6 desaturases to silkworm by mulberry leaves could make silkworm a new source of these FAs, an efficient and affordable replacement for fish oils. 

Although certain inconveniences were reported when used in animal feeding, SP generally had a beneficial impact on performance, meat quality, and egg quality. The amount of waste that must be discarded is reduced when SP is used to feed people and animals. One of the techniques to reduce pollution emissions consists of performing this. On the other hand, mulberry leaves are a unique type of feed for the silkworm *B. mori* and can sequester carbon. The mulberry trees contributes significantly to the absorption of harmful pollutants. Research on silkworms may make it easier to investigate insect domestication, morphogenesis, endocrinology, reproduction, behavior, and immunology. It may also help to clarify the role of gene homologs, being used as a model organism for medicinal purposes.

## Figures and Tables

**Table 1:** Nutritional composition of silkworm pupae (*B. mori*).

Items	% (DM Bases)	Reference
Protein	48–60	Herman et al. [22]
	49–54	Wu et al. [19]
	59.8–75.1	Shukurova et al. [28]
	52–80	Zotte et al. [29]
	59.52–94.98	Lamberti et al. [21] *
	55.60	Kumar et al. [23]
	49–54	Nowak et al. [30]
	55.6	Tomotake et al. [24]
	49.1–53.5	Pereira et al. [31]
	48.7–51.6	Singh and Jayasomu [32]
	58.8	Feedpedia [33]
	48.7	Rumpold et. al. [34]
Fat	30	Herman et al. [22]
	25–30	Wu et al. [19]
	12.1–27.4	Shukurova et al. [28]
	29	Kouřimská and Adámkov [25]
	32.2	Kumar et al. [23]
	32.2	Tomotake et al. [24]
	33.3–35.7	Pereira et al. [31]
	30	Singh and Jayasomu [32]
	28.5	Feedpedia [33]
	30.10	Rumpold et. al. [34]
Fiber	3.5–4.7	Shukurova et al. [28]
	14	Kouřimská and Adámkov [25]
	5.8	Feedpedia [33]
Energy	5.09–6.82 MJ/kg	Lamberti et al. [21]
	26.5 MJ/kg DM	Feedpedia [33]

* Calculated to express concentration as DM bases.

**Table 2:** Centesimal fatty acids composition of total lipids in SP.

Fatty Acids (%)	Male	Female	Total	Reference
C16:0 (palmitic)	28.60	22.80		Nakasone and Ito [43]
	24.90	19.50		Kotake-Nara et al. [39]
			21.3–28.30	Pereira et al. [31]
			24.20	Tomotake et al. [24]
			23.18	Kumar et al. [5]
			23.18	Zhou et al. [17]
C16:1n-7 (palmitoleic)	3.10	1.80		Nakasone and Ito [43]
	0.80	0.60		Kotake-Nara et al. [39]
			0.60–0.70	Pereira et al. [31]
			1.70	Tomotake et al. [24]
			1.07	Kumar et al. [5]
			1.07	Zhou et al. [17]
C18:0 (stearic)	2.60	4.30		Nakasone and Ito [43]
	5.40	6.30		Kotake-Nara et al. [39]
			5.5–9.20	Pereira et al. [31]
			4.50	Tomotake et al. [24]
			4.69	Kumar et al. [5]
			4.69	Zhou et al. [17]
C18:1n-9 (oleic)	29.0	27.20		Nakasone and Ito [43]
	24.30	22.60		Kotake-Nara et al. [39]
			30.6–38.0	Pereira et al. [31]
			26.00	Tomotake et al. [24]
			28.32	Kumar et al. [5]
			28.32	Zhou et al. [17]
C18:2n-6 (linoleic)	7.30	8.50		Nakasone and Ito [43]
	6.30	7.70		Kotake-Nara et al. [39]
			5.81–8.57	Pereira et al. [31]
			7.30	Tomotake et al. [24]
			3.88	Kumar et al. [5]
			3.88	Zhou et al. [17]
C18:3n-3 (alpha-linolenic)	29.20	34.90		Nakasone and Ito [43]
			17–33.40	Pereira et al. [39]
	36.00	40.70		Kotake-Nara et al. [31]
			36.30	Tomotake et al. [24]
			38.25	Kumar et al. [5]
			38.25	Zhou et al. [17]

## Data Availability

The data presented in this study are available on request from the corresponding author.
